# Toxigenic *Corynebacterium diphtheriae*–Associated Genital Ulceration

**DOI:** 10.3201/eid2609.180830

**Published:** 2020-09

**Authors:** Frieder Fuchs, Derya Markert, Isabel V. Wagner, Max C. Liebau, Anja Berger, Alexandra Dangel, Andreas Sing, Mario Fabri, Georg Plum

**Affiliations:** Institute for Medical Microbiology, Immunology and Hygiene, University of Cologne, Medical Faculty and University Hospital of Cologne, Cologne, Germany (F. Fuchs, D. Markert, G. Plum);; University of Cologne, Medical Faculty and University Hospital of Cologne, Cologne (I.V. Wagner, M.C. Liebau, M. Fabri);; Center for Molecular Medicine Cologne, University of Cologne, Faculty of Medicine, Cologne (M.C. Liebau);; Bavarian Health and Food Safety Authority, National Consulting Laboratory for Diphtheria, Oberschleißheim, Germany (A. Berger, A. Dangel, A. Sing)

**Keywords:** Sexually transmitted infections, sexually transmitted diseases, child refugee, cutaneous diphtheria, genital ulcer disease, bacteria, Corynebacterium diphtheriae, Germany, Ethiopia

## Abstract

In October 2016, an adolescent boy sought care for acute genital ulceration in Cologne, Germany. We presumed a sexually transmitted infection, but initial diagnostic procedures yielded negative results. He was hospitalized because swab samples from the lesion grew toxigenic *Corynebacterium diphtheriae*, leading to the diagnosis of possibly sexually transmitted cutaneous diphtheria.

Members of the genus *Corynebacterium* colonize the upper respiratory tract and healthy skin and are frequently found in clinical specimens without clinical significance. Three species, *C. diphtheriae*, *C. ulcerans*, and *C. pseudotuberculosis*, can cause serious infections by acquiring the ability to produce the diphtheria toxin manifesting clinically as cutaneous or respiratory diphtheria. Because vaccination is widely used in industrialized countries, diphtheria is rare, but it remains endemic to several countries of Africa, Asia, the Eastern Pacific region, and Eastern Europe. During 2001–2016, a total of 80 cases of toxigenic *Corynebacterium* spp. infections were notified in Germany (*1*).

## Case Report

In October 2016, a 16-year-old boy from Ethiopia sought care at the University Hospital of Cologne (Cologne, Germany) with 3 papules at the glans and a slightly bleeding, painful ulceration at the dorsal shaft of his penis. He complained about local pruritus and had swollen, painful inguinal nodules on the left side. On clinical examination, the ulcer appeared to have coalesced from a few smaller lesions with an erythematous base and a yellow to purulent exudate. The boy denied having lesions before the ulcer. Specific history could not be clarified satisfactorily: the boy had an undefined status of residence and lived in an accommodation facility for asylum seekers. Given that he was an unaccompanied minor refugee, the examiners considered traumatic sexual experiences when the boy was unwilling to share further details especially about his sexual history. General physical examination was unremarkable. Pubertal development was normal.

We presumed a sexually transmitted infection. Because we considered herpes simplex virus and chancroid as likely diagnoses, the boy started on empiric outpatient treatment with valaciclovir and azithromycin. Serologic screening for syphilis and herpes simplex virus PCR yielded negative results. Toxigenic *C. diphtheriae* was cultured from the ulcer, and the boy was hospitalized 7 days after initial consultation because of cutaneous diphtheria. Toxigenicity of the isolate was verified by real-time PCR for *tox* gene and the modified Elek test in the German National Consulting Laboratory for Diphtheria. On the day of hospitalization the lesions showed only minor signs of resolution. Blood test analysis did not detect any systemic inflammation. No other swab of the genital lesions or the uninflammed tonsils grew pathogens. Because the boy’s vaccination history was unclear, we used an enzyme immunoassay to determine the serum level of diphtheria toxin antibodies, and the results indicated seroprotection (4.69 IU/mL).

The patient was isolated and treated with intravenous penicillin (6 million U/d). When susceptibility testing revealed penicillin resistance, his antimicrobial drug regimen was changed to ciprofloxacin (1,200 mg/d). After 4 days of inpatient treatment, the patient was discharged. Follow-up visits confirmed full recovery; repeat swab sample testing of throat and penis were culture- and PCR-negative. The patient completed antimicrobial drug therapy 10 days after discharge.

In contact tracing, initiated by the local health department, 2 persons were determined to be at risk because they shared a room with the patient at the accommodation facility. Swab samples from tonsils and suspicious skin lesions (including 1 penile wound) of the contacts were negative for *C. diphtheriae*. Consequently, we discussed public health actions with them; chemoprophylaxis or vaccination were not considered necessary.

## Conclusions

Only few cases of cutaneous diphtheria are reported in Germany, and they are strongly associated with defined risk factors, especially contact, during endemic circulation, associated with traveling (*2*), migration (*3*), zoonotic reservoirs (*4*), or poor socioeconomic conditions (*5*). In this case, the clinical manifestion of the genital ulcer with typical symptoms of sexually transmitted infections, such as painful unilateral inguinal nodules, supports the likelihood of sexually transmitted diphtheria, although we were not able to verify the patient’s medical or sexual history. We did not isolate any other pathogen, in contrast with another case of male urethritis in which toxigenic *C. diphtheriae* was part of a polymicrobial infection and sexual transmission was assumed (*6*). This case shows that unusual manifestations of diphtheria are conceivable on various body sites. Clinicians should consider this easily overlooked disease in the differential diagnosis for similar cases. This awareness might lead to an increase in confirmed cases, improve outcomes, and prevent disease through subsequent public health actions.
